# Height and timing of growth spurt during puberty in young people living with vertically acquired HIV in Europe and Thailand

**DOI:** 10.1097/QAD.0000000000002294

**Published:** 2019-07-09

**Keywords:** Europe, growth, height, HIV, perinatal, puberty, Thailand

## Abstract

The aim of this study was to describe growth during puberty in young people with vertically acquired HIV.

**Design:**

Pooled data from 12 paediatric HIV cohorts in Europe and Thailand.

**Methods:**

One thousand and ninety-four children initiating a nonnucleoside reverse transcriptase inhibitor or boosted protease inhibitor based regimen aged 1–10 years were included. Super Imposition by Translation And Rotation (SITAR) models described growth from age 8 years using three parameters (average height, timing and shape of the growth spurt), dependent on age and height-for-age *z*-score (HAZ) (WHO references) at antiretroviral therapy (ART) initiation. Multivariate regression explored characteristics associated with these three parameters.

**Results:**

At ART initiation, median age and HAZ was 6.4 [interquartile range (IQR): 2.8, 9.0] years and −1.2 (IQR: −2.3 to −0.2), respectively. Median follow-up was 9.1 (IQR: 6.9, 11.4) years. In girls, older age and lower HAZ at ART initiation were independently associated with a growth spurt which occurred 0.41 (95% confidence interval 0.20–0.62) years later in children starting ART age 6 to 10 years compared with 1 to 2 years and 1.50 (1.21–1.78) years later in those starting with HAZ less than −3 compared with HAZ at least −1. Later growth spurts in girls resulted in continued height growth into later adolescence. In boys starting ART with HAZ less than −1, growth spurts were later in children starting ART in the oldest age group, but for HAZ at least −1, there was no association with age. Girls and boys who initiated ART with HAZ at least −1 maintained a similar height to the WHO reference mean.

**Conclusion:**

Stunting at ART initiation was associated with later growth spurts in girls. Children with HAZ at least −1 at ART initiation grew in height at the level expected in HIV negative children of a comparable age.

## 1 Introduction

Although young people living with HIV are at risk for poor height growth [1], treatment with antiretroviral therapy (ART) improves growth, with strongest gains in those treated at a young age [2]. Although initial catch-up growth on ART has been well described [2], there are less data on long term growth, particularly during adolescence.

Delays in pubertal development have been reported in young people with HIV [3–7], with the onset of puberty [5] and sexual maturation [6] occurring 6 months later compared with HIV-exposed uninfected young people (HEU). Earlier puberty in the general population is associated with being taller and having higher BMI throughout childhood [8], and poor growth in children with HIV has been shown to account for much of the delay in reaching sexual maturity [6]. There is also evidence that children starting ART with low height-for-age *z*-scores experience delays in the onset of puberty independently of age at ART initiation [3].

Poor growth during childhood can have implications for future health. Height velocity is associated with increased HIV replication [9] and progression to AIDS and death [10] with the association with death being independent of age, viral load and CD4^+^ cell count [11]. The timing of puberty is also inversely associated with bone mass and density among HIV-negative adolescents [12] and delayed puberty may increase future risk of osteoporosis among young people with HIV, who themselves are at risk of poor bone health, either caused by HIV infection itself or prolonged exposure to ART [13]. Early growth failure has also been linked to poorer social and economic outcomes in later life in the general population [14].

In this study, statistical models that describe an individual's growth in terms of mean height throughout adolescence, and timing and shape of the adolescent growth spurt were applied to longitudinal height measurements. The overall aim of this study was to explore the association between characteristics at ART initiation, in particular age and height-for-age *z*-score, and growth during adolescence.

## 2 Materials and methods

Seventeen paediatric HIV cohorts from 15 countries contributed individual level data to the European Pregnancy and Paediatric HIV Cohort Collaboration (EPPICC) between September 2016 and March 2017 using a modified HICDEP protocol (www.hicdep.org). Pseudo-anonymized data on all children at participating clinics were included. All cohorts received approval from local and/or national ethical committees. Five cohorts from three countries (Italy, Ukraine and three from Russia) where height data were not routinely collected (each with <20% of children having a height measurement at ART initiation) were excluded. Children from the remaining 12 cohorts were eligible provided they initiated ART with at least two nucleoside reverse transcriptase inhibitors (NRTIs) along with a nonnucleoside reverse transcriptase inhibitor (NNRTI) or boosted protease inhibitor (bPI); were 1–10 years old at ART initiation; not known to have horizontally acquired HIV; and aged at least 8 years at the end of follow-up. We excluded children initiating ART after age 11 years. For those initiating ART at an older age, it would be difficult to distinguish between changes in growth occurring as a result of a pubertal growth spurt and as a result of initiating ART. Children with no height recorded at ART initiation and/or after 8 years of age were excluded.

Height measurements were censored at the earliest of 19th birthday, transfer to adult care, death or loss to follow-up. Height and BMI were converted to height-for-age *z*-scores (HAZ) and BMI-for-age *z*-scores (zBMI), using the WHO Growth Standard for measurements when children were aged under 5 years [15] and the WHO 2007 growth reference when aged 5–18 years [15,16]. Data checks were carried out to detect implausible changes in height and/or HAZ. HAZ was categorized according to WHO definitions as less than −3 SD (severe stunting); −3 to less than −2 SD (stunting); −2 to less than −1 SD; and at least −1 SD. zBMI was categorized as less than −2 SD (underweight); −2 to 1 SD (normal); more than 1 to 2 SD (overweight); and more than 2 SD (obese). HAZ and zBMI nearest to ART initiation (closest within 6 months before to 1 month after) were considered baseline measurements.

Other variables included were sex, country (Thailand, UK/Ireland, Rest of Europe), age at ART initiation (1–2, 3–5 or 6–10 years), initial ART regimen (protease inhibitor, NNRTI), being born outside the country of the cohort (’born abroad’) and immunodeficiency at ART initiation classified using the WHO immunological classification: none (CD4% >35%, >30%, >25% or CD4^+^ cell count >500 cells/μl in children <1, 1–2, 3–4 and ≥5 years, respectively), mild (CD4% 30–35%, 25–30%, 20–25% or CD4^+^ cell count 350–499 cells/μl), advanced (CD4% 25–29%, 20–24%, 15–19% or CD4^+^ cell count 200–349 cells/μl) or severe (CD4% <25%, <20%, 15–19% or in children >5 CD4^+^ cell count <200 cells/μl or CD4% <15%) [17].

### 2.1 Statistical analysis

Characteristics at ART initiation were summarized by HAZ category. Mean height at age 16 years was summarized by age and HAZ at ART initiation and compared with the WHO reference height to quantify differences in height following the growth spurt. It was not possible to assess differences in final height, as many adolescents transfer to adult care from age 16 years, ending follow-up in EPPICC.

Height was modelled using Super Imposition by Translation And Rotation (SITAR) models [18]. SITAR was developed to model growth during childhood and adolescence and quantifies differences in growth via three parameters representing the timing and shape of the adolescent growth spurt, as well as average height. The models can explain up to 99% of the variation between individuals’ growth [18] and can be summarized as: 
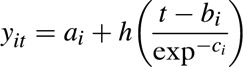


where the outcome *y*_*it*_ is the height of individual *i* at age *t* and *h*( ) is a natural cubic spline of height over age. The parameters *a_i_*, *b_i_* and *c_i_* are participant specific random effects. *a_i_* represents average height throughout adolescence; negative values indicate shorter height overall. *b_i_* represents timing of the pubertal growth spurt; negative values indicate earlier puberty. *c_i_* represents growth velocity, or the shape of the growth spurt; positive values indicate shorter growth spurts and a steeper growth velocity curve, while negative values indicate the growth spurt occurs over a longer duration. Corresponding growth velocity curves can also be estimated as the first derivative of the modelled growth (height) curve.

Age at peak height velocity (APHV) is correlated with timing of puberty and often used as a proxy for timing of maturation. It commonly occurs in girls in Tanner stage 2 or 3 and in Tanner stage 3 or 4 for boys [19,20], though there is variation in timing across Tanner stages [19]. Differences in the timing of the growth spurt estimated using SITAR models have been shown to be highly correlated with APHV [18].

All height measurements (in cm) from age 8 (or start of ART if after 8th birthday) to 18 years were included. Age and HAZ at ART initiation were added to the SITAR model as fixed effects that could influence the mean of *a, b* and *c*. Thus, the estimated random effects *a_i_*, *b_i_* and *c_i_* represent the individual differences in average height, timing and shape of the growth spurt not associated with differences in age or height at ART initiation. Models were fitted separately to boys and girls using a spline with 6 degrees of freedom. Log transformations of both age and height [18] were considered, but the untransformed data provided the best fit. Interactions between baseline height and age were added where appropriate [model comparison carried out using Bayes Information Criteria (BIC)].

To explore other factors (sex, country, initial ART regimen, WHO immunological classification, zBMI at ART initiation) associated with growth after allowing for differences in baseline age and height, the estimated *a_i_*, *b_i_* and *c_i_* random effects from the SITAR model were analysed using multivariable linear regression. Interactions between each of the factors and sex and between immunological classification and HAZ and age at ART initiation were considered. A second model was fitted including zBMI at age 8 years instead of at ART initiation.

Modelling was repeated in countries where more than 5% of children were born abroad and more than 5% born in the country (UK and Ireland, Spain and Netherlands) to explore differences between those born abroad and those born in the cohort country. Three sensitivity analyses were carried out: in the first separate models were fitted for children from Thailand and elsewhere; in the second Thai-specific growth reference data were used for Thai children [21]; and in the third children starting ART after their eighth birthday were excluded.

Analyses were carried out using Stata statistical software release 15 (StataCorp LP, College Station, Texas, USA) and the SITAR package [22] in R v3.3.3 (R Core Team, R Foundation for Statistical Computing, Vienna, Austria).

## 3 Results

### 3.1 Patient characteristics

**Fig. 1:**
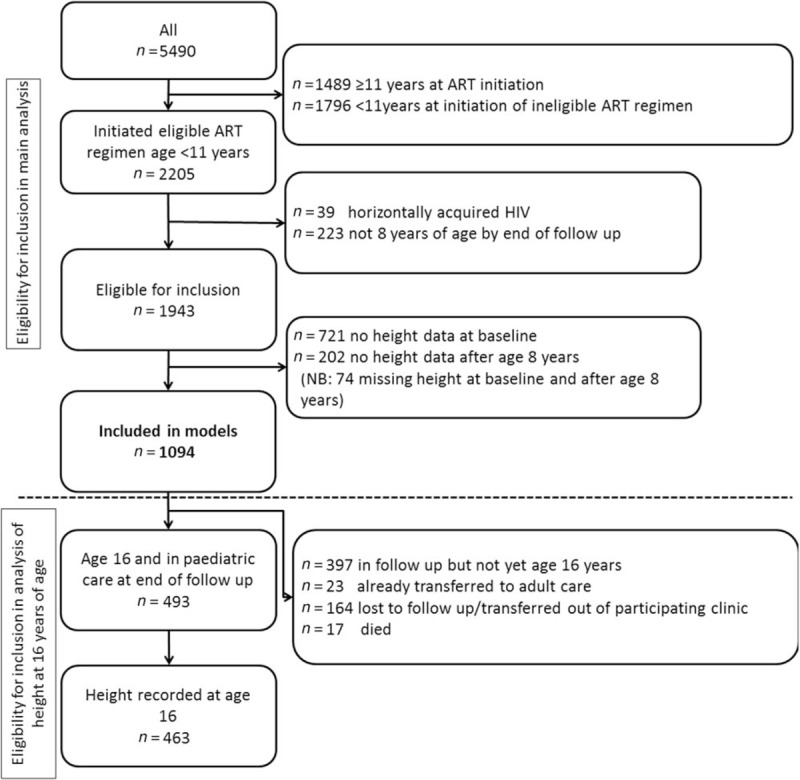
Flow chart of participants included in the study.

In total, 1943 young people with HIV initiated ART on an eligible regimen age 1–10 years and were at least 8 years old at the end of follow-up (Fig. 1). After excluding those with missing baseline height (*n* = 721) and/or height after age 8 years (*n* = 202), we included 1094 children in the analysis. Children excluded due to missing height data were more likely to be from countries other than Thailand or UK/Ireland, be born abroad and be younger at ART initiation than those who were included (Supplementary Table 1). The 1094 included children were followed-up for a median of 9.1 (6.9, 11.4) years after ART initiation. During this time, 37 325 height measurements were recorded with a median of 32 (19, 46) per child, of which 25 458 [median 21 (11, 32)] were from age 8 years onwards. The median time between height measurements was 2.8 (1.4, 3.9) months, with some variation by cohort ranging from every 1.8 (1.0, 2.8) months in Thailand to 8.3 (4.7, 11.3) months in Greece.

At ART initiation, median HAZ was −1.2 (−2.3, −0.2) and age was 6.4 (2.8, 9.0) years. Characteristics of children at ART initiation, stratified by baseline HAZ, are described in Table 1. More severe stunting was associated with residence in Thailand, not being born abroad, initiating on an NNRTI based regimen, earlier calendar year of ART initiation, higher viral load, more severe immunodeficiency and lower zBMI at ART initiation.

At the end of the study, 493 (45%) children had reached their 16th birthday while still in paediatric care (Fig. 1), of whom 463 (94%) had their height recorded within 6 months of their birthday. Children who survived to age 16 years but were no longer in follow-up in paediatric care were more likely to reside in Thailand and start ART at a younger age. At age 16 years, the mean (standard deviation) heights of boys and girls were 166 (8.7)cm and 158 (6.9)cm, respectively, significantly shorter than the WHO reference mean height of 173 (7.8)cm for boys and 163 (6.8)cm for girls (both *P* < 0.001) (Supplementary Table 2).

### 3.2 Associations between age and height-for-age *z*-score at antiretroviral therapy initiation and growth from age 8 years

Results from the SITAR models are available in Supplementary Table 3. Estimated mean height and corresponding growth velocity curves stratified by HAZ and by age are summarized in Fig. 2 a and b, respectively, for girls and Fig. 3 a and b for boys.

**Fig. 2:**
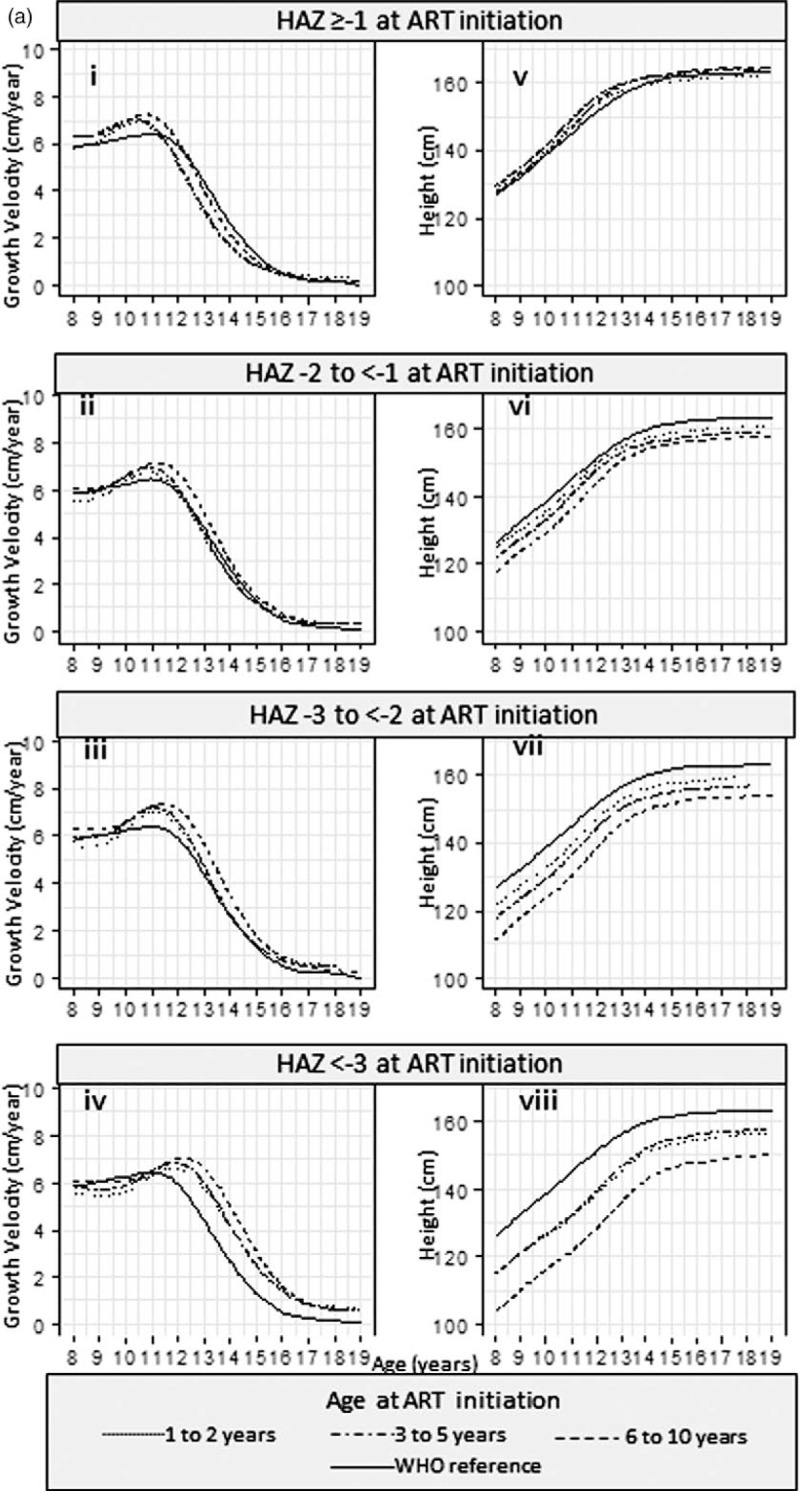
(a) Mean height and growth velocity of girls stratified by HAZ at ART initiation and (b) mean height and growth velocity of girls stratified by age at ART initiation.

**Fig. 2 (Continued):**
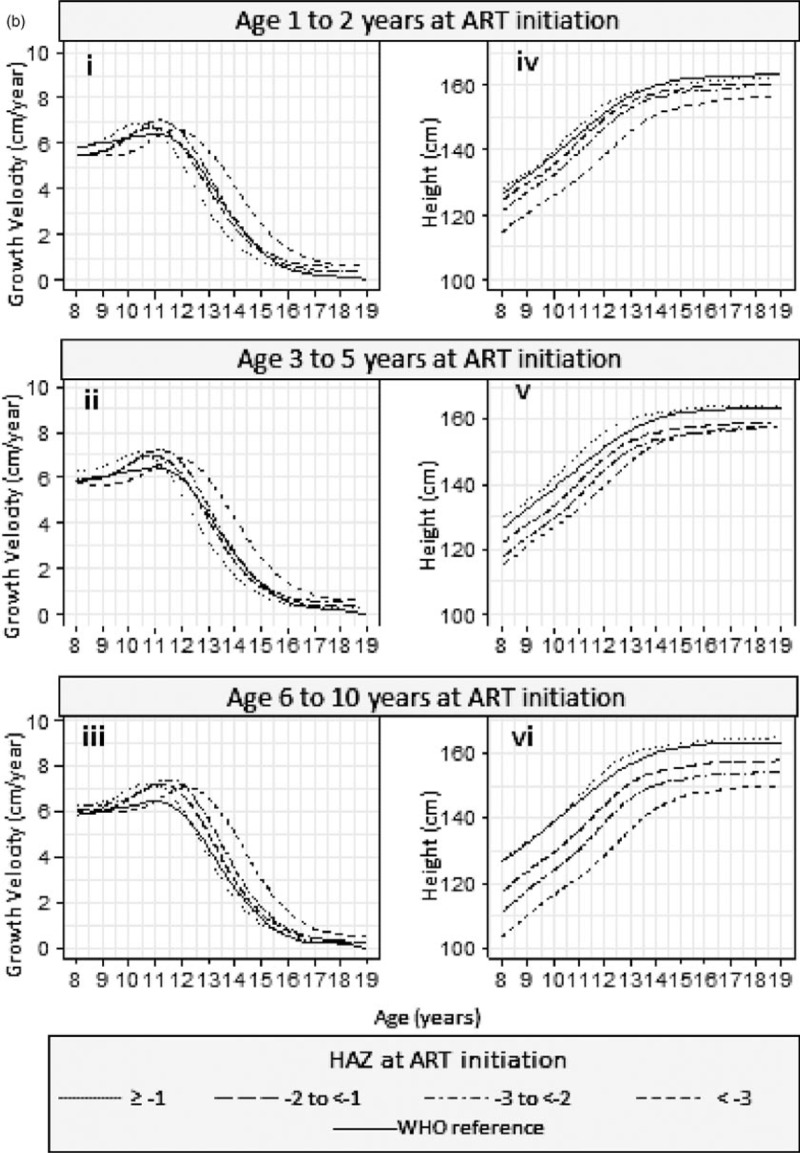
(a) Mean height and growth velocity of girls stratified by HAZ at ART initiation and (b) mean height and growth velocity of girls stratified by age at ART initiation.

**Fig. 3:**
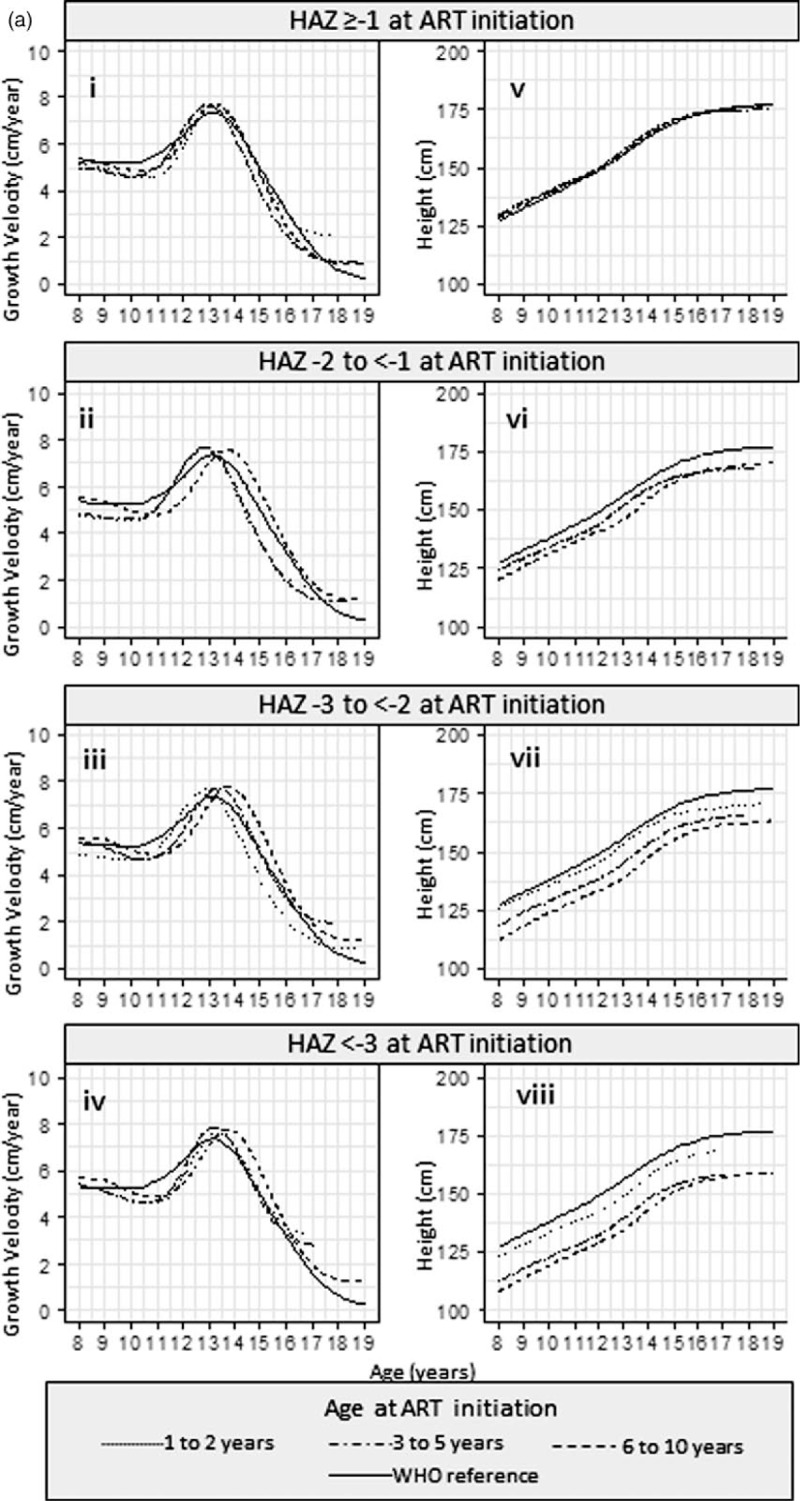
(a) Mean height and growth velocity of boys stratified by HAZ at ART initiation and (b) mean height and growth velocity of boys stratified by age at ART initiation.

**Fig. 3 (Continued):**
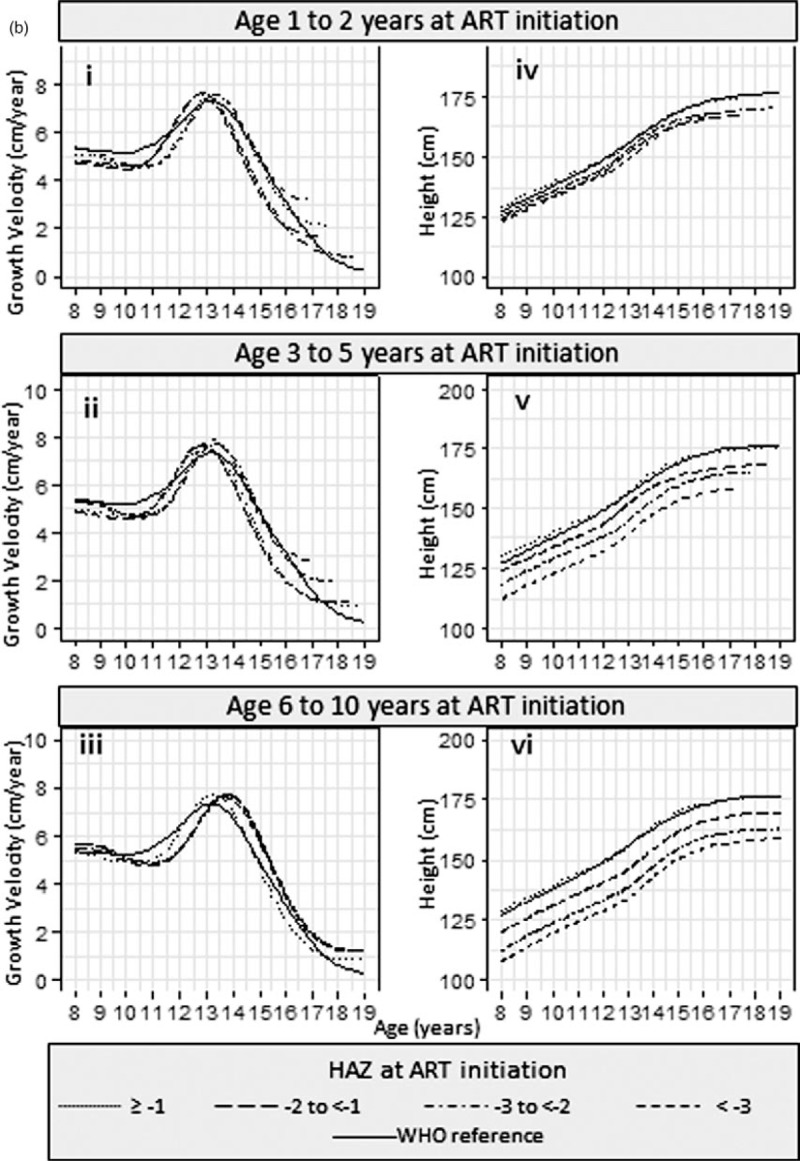
(a) Mean height and growth velocity of boys stratified by HAZ at ART initiation and (b) mean height and growth velocity of boys stratified by age at ART initiation.

In girls, across each of the baseline HAZ groups (Fig. 2ai–iv), children starting ART in the oldest age group had growth spurts on average 0.41 [95% confidence interval (95% CI) 0.20–0.62] years later than those starting ART in the youngest age group. Across the baseline age groups (Fig. 2bi–iii), girls starting ART with low HAZ had later growth spurts; there was a 1.50 (1.21–1.78) year delay in those with baseline HAZ less than −3 compared with baseline HAZ at least −1. The effect of this delay on overall height can be seen in Fig. 2 b iv-vi; the differences in height are smaller from age 16 onwards (after the growth spurt) than at age 8 years.

In boys, the association between baseline age and the timing of the growth spurt differed by baseline HAZ (Fig. 3 ai–iv); there was no significant difference by age in boys who started ART with HAZ at least −1 (Fig. 3 ai). In boys with baseline HAZ of −2 to less than −1 (Fig. 3 aii), the growth spurt was 0.96 (0.19–1.72) years later in those starting ART in the oldest compared with the youngest age group. Similarly, for a baseline HAZ of −3 to less than −2 (Fig. 3 aiii), the corresponding delay in those starting ART in the oldest age group was 0.92 (0.17–1.66) years, and for baseline HAZ less than −3, it was 0.42 (−0.32 to 1.16) years (Fig. 3 aiv). The timing of the growth spurt in boys did not differ significantly by baseline HAZ (Fig. 3 bi–iii).

Girls (Fig. 2 bv) and boys (Fig. 3 bv), who started treatment with a baseline HAZ at least −1, maintained a similar mean height to the WHO reference, regardless of baseline age.

### 3.3 Other factors associated with growth from age 8 years

Characteristics associated with variations in growth that remained after adjusting for differences in baseline HAZ and age are summarized in Table 2. Young people from Thailand were smaller throughout adolescence than those from other countries, but did not differ in the timing of the growth spurt. The shape of the growth spurt differed by country and was shorter in children from the UK and Ireland than elsewhere. Lower zBMI at ART initiation was significantly associated with a later growth spurt [a one SD decrease was associated with a 0.07 (0.02–0.11) year delay in the growth spurt]. In a second model (data not shown), a one SD decrease in zBMI at age 8 years was associated with a 0.16 (0.09–0.22) year delay in the timing of the growth spurt, while other parameters did not change substantially. There was no evidence of any interactions.

In subgroup analysis (*n* = 545), there was a significant interaction between sex and being born abroad on timing of the growth spurt (*P* = 0.038). Girls born abroad experienced a growth spurt 0.24 (0.02–0.46) years earlier than those born in the cohort country, although there was no association in boys. However, after adjusting for zBMI at age 8, the association was no longer significant [growth spurt for girls born abroad was 0.18 (−0.05 to 0.42) years earlier].

In the three sensitivity analyses wherein models were fitted separately to children from Thailand and elsewhere, Thai-specific reference data were used for Thai children and children starting ART age at least 8 years were excluded, overall conclusions were unchanged (data not shown).

## 4 Discussion

In this study, we described growth throughout adolescence in a large cohort of young people with vertically acquired HIV in Europe and Thailand. Although all adolescents in the study initiated ART before age 11 years, growth deficits remained throughout adolescence. Only children with HAZ at least −1 when starting ART were able to achieve a similar height to the WHO reference at age 16 years, suggesting that for others, catch up growth associated with being on ART long term was not sufficient to restore height to what would be expected in an HIV-negative population.

We observed an association between older age at ART initiation and later growth spurts in boys (with HAZ <−1 at ART initiation) and girls, in line with findings from the Antiretroviral research for Watoto (ARROW) trial wherein attainment of each tanner stage and onset of menarche was delayed in those starting ART at older ages [3]. We also observed an association between stunting and later growth spurts, but only in girls. The potential role of anthropometric parameters in early childhood on growth during puberty was highlighted in a study of 2539 young people with vertically acquired HIV and HIV-exposed uninfected (HEU) young people from the USA [6]. Young people living with HIV reached sexual maturity on average 6 months later than the HEU group, but differences in HAZ prior to puberty accounted for up to 98% of the delay in boys and (together with zBMI) 74% in girls, suggesting much of the delay may be attributable to earlier poor growth [6]. Low HAZ at ART initiation was also associated with delayed attainment of all Tanner stages in boys and girls, and menarche in girls, independently of age at ART initiation in the ARROW trial [3]. However, in boys, the delay was reduced in those who had the greatest initial gains in CD4^+^ cell count after starting ART, but there was no similar association in girls. Undernutrition early in life was also found to have a stronger association with adult height in women than men in the Netherlands [23]. Although this suggests that girls may be more sensitive to impairments early in life, and prior to ART, the mechanism underlying potential sex differences remain to be explained.

After accounting for HAZ and age at ART initiation, we found no association between WHO immunological status or viral load at ART initiation and growth. Similarly, the ARROW trial found immune suppression prior to ART was not associated with delayed puberty or menarche [3]. Other studies have also reported a lack of association between clinical status at start of puberty and age at onset [4,7]. However, in young people in the USA, low CD4^+^ cell count and high viral load at first pubertal assessment were associated with later pubertal onset. Among boys, prior CDC C, low nadir CD4% or high peak viral load were also associated with later puberty [5]. However, many of these young people initiated ART on mono or dual therapy and are likely to have substantially different treatment histories compared with our study.

We found zBMI at ART initiation and age 8 to be associated with the timing of the growth spurt, with no evidence of a difference between boys and girls. We also observed that girls born abroad experienced an earlier pubertal growth spurt than girls born in the cohort country, but the differences in the timing of the growth spurt reduced after adjusting for zBMI at age 8. In girls, a relationship between low BMI and delayed puberty has been found in multiple studies [8] and rapid weight gain prior to puberty also linked to early onset [24]. Differences between young people born abroad and those born in the country may therefore be explained by periods of more rapid weight gain in children arriving from abroad, the majority from Africa, compared with those born in the country.

This study had several limitations; as with all observational studies, our findings on the association between age and HAZ at ART initiation and growth should not be over interpreted or assumed to be causative. At ART initiation, stunting was strongly correlated with immunosuppression, viral load and zBMI and may be a marker for poor immunological status and other impairments. Children starting ART at older ages represent a group who have survived without treatment and possibly with limited access to care and so may be subject to a survivor bias. Had ART initiation been delayed in those who started at a young age, the observed delay in the growth spurt associated with starting ART at an older age may have been less in this group who would also have been more likely to have access to healthcare and regular monitoring. Nonetheless, the findings provide insight in to growth patterns among children presenting to care and starting ART at different ages.

Inclusion criteria applied also lead to the potential for selection bias. We excluded children with missing height data. Multiple imputation was not possible, as other data, such as immunological and virological status, at ART initiation, likely to be strong predictors of baseline height were missing in more than half of the children with missing heights. We excluded young people from Russia, Ukraine and Italy where height data were not routinely recorded. Further, the cohorts included in EPPICC range from national coverage to city hospitals leading to potential for bias where children treated in large city hospitals are not representative of others in the country. Our analyses were restricted to children aged 1–10 years at ART initiation. The number of infants initiating ART under age 1 year was small, with high rates of missing baseline data. A further limitation is the lack of quantitative measures of pubertal status such as Tanner stage and date of onset of menarche, which is not routinely collected by the majority of participating cohorts. However, differences in timing of the growth spurt are likely to be indicative of differences in the timing of onset of puberty.

Finally, we used the WHO growth standard [15] and growth reference [16] to derive *z*-scores at ART initiation. Although the WHO growth standards were developed to assess growth globally, children from Thailand were significantly shorter than those residing in Europe and the WHO reference may overestimate stunting as compared to Thailand's own national growth reference [25]. However, in sensitivity analyses, using Thai reference data, we did not find any difference in the associations between baseline HAZ and growth during adolescence.

Despite these limitations, the study has several strengths. The collaborative nature of the study provides a rich source of longitudinal height measurements from a large sample of young people living with HIV followed during childhood and adolescence and the use of SITAR models provides insight into growth during puberty in the absence of quantitative measures of pubertal status.

In summary, we have shown that children who initiate ART at younger ages are taller. Children who initiate ART with a ‘normal’ height for age *z*-score (HAZ ≥−1) remained with a ‘normal’ height throughout adolescence. Those who initiated ART stunted or severely stunted were less likely to achieve ‘normal’ height. We also demonstrated that in girls, regardless of age at ART initiation, stunting at time of initiation was associated with a later pubertal growth spurt, and this continued growth into later adolescents may allow those most severely stunted to catch-up somewhat. However, longer-term follow-up is required to understand the potential implications of delayed pubertal growth on outcomes in later life.

## 5 Acknowledgements

We thank all the patients for their participation in these cohorts, and the staff members who cared for them.

Author contributors: Writing Group (consisting of Project Team first (ordered alphabetically by name except for the first and last author), and also other Writing Group members (ordered alphabetically by cohort name)):

Project Team: Siobhan Crichton (EPPICC statistician), Eric Belfrage (Karolinska Institutet and University Hospital, Stockholm, Sweden), Intira Jeanne Collins (Collaborative HIV Paediatric Study (CHIPS), UK and Ireland), Katja Doerholt (Collaborative HIV Paediatric Study (CHIPS), UK and Ireland), Ali Judd (co-lead of EPPICC), Sophie Le Coeur (Thailand Program for HIV Prevention and Treatment (PHPT) Study Group, Thailand), Vana Spoulou (Greece Cohort, Greece), Ruth Goodall (EPPICC senior statistician).

Other Writing Group members: Henriette Scherpbier, Colette Smit (ATHENA paediatric cohort, Netherlands); Tessa Goetghebuer (Hospital St Pierre Cohort, Brussels, Belgium); Diana M. Gibb (Collaborative HIV Paediatric Study (CHIPS) and National Study of HIV in Pregnancy and Childhood (NSHPC), UK & Ireland); Antoni Noguera (CoRISPE-CAT, Catalonia cohort, Spain) Maria Luisa Navarro, Jose Tomas Ramos (CoRISPE-S, rest of Spain cohort, Spain); Luisa Galli (Italian Register for HIV infection in children, Italy); Carlo Giaquinto, Claire Thorne (Paediatric European Network for the Treatment of AIDS (PENTA), Italy); Santa Ansone (Latvian cohort, Latvia); Magdalena Marczynska (Polish paediatric cohort, Poland); Liubov Okhonskaia (Republican Hospital of Infectious Diseases, St Petersburg, Russia); Begoña Martinez de Tejada (Swiss Mother and Child HIV Cohort Study, Switzerland); Gonzague Jourdain, Luc Decker (Thailand Program for HIV Prevention and Treatment (PHPT) Study Group, Thailand); Luminita Ene (’Victor Babes’ Hospital Cohort, Romania).

**COLLABORATING COHORTS**

**Belgium:** Hospital St Pierre Cohort, Brussels: Tessa Goetghebuer, MD, PhD; Marc Hainaut, MD PhD; Evelyne Van der Kelen, Research nurse; Marc Delforge, data manager.

**Greece:** Greek cohort: Vana Spoulou.

**Italy:** Italian Register for HIV infection in Children. Coordinators: Maurizio de Martino (Florence), Pier Angelo Tovo (Turin). Participants: Osimani Patrizia (Ancona), Domenico Larovere (Bari), Maurizio Ruggeri (Bergamo), Giacomo Faldella, Francesco Baldi (Bologna) Raffaele Badolato (Brescia), Carlotta Montagnani, Elisabetta Venturini, Catiuscia Lisi (Florence), Antonio Di Biagio, Lucia Taramasso (Genua), Vania Giacomet, Paola Erba, Susanna Esposito, Rita Lipreri, Filippo Salvini, Claudia Tagliabue (Milan), Monica Cellini (Modena), Eugenia Bruzzese, Andrea Lo Vecchio (Naples), Osvalda Rampon, Daniele Donà (Padua), Amelia Romano (Palermo), Icilio Dodi (Parma), Anna Maccabruni (Pavia), Rita Consolini (Pisa), Stefania Bernardi, Hyppolite Tchidjou Kuekou, Orazio Genovese (Rome), Paolina Olmeo (Sassari), Letizia Cristiano (Taranto), Antonio Mazza (Trento), Clara Gabiano, Silvia Garazzino (Turin), Antonio Pellegatta (Varese)

**Latvia:** Latvian Cohort (Santa Ansone).

**Netherlands:** The ATHENA database is maintained by Stichting HIV Monitoring and supported by a grant from the Dutch Ministry of Health, Welfare and Sport through the Centre for Infectious Disease Control of the National Institute for Public Health and the Environment.

**CLINICAL CENTRES (PAEDIATRIC CARE)**

**Emma Kinderziekenhuis, Amsterdam, University Medical Centers:***HIV treating physicians:* D. Pajkrt, H.J. Scherpbier. *HIV nurse consultants:* A.M. Weijsenfeld, C.G de Boer. *HIV clinical virologists/chemists:* S. Jurriaans, N.K.T. Back, H.L. Zaaijer, B. Berkhout, M.T.E. Cornelissen, C.J. Schinkel, K.C.wolthers. **Erasmus MC–Sophia, Rotterdam:***HIV treating physicians:* P.L.A. Fraaij, A.M.C. van Rossum, C.L. Vermont. *HIV nurse consultants:* L.C. van der Knaap, E.G. Visser. *HIV clinical virologists/chemists:* C.A.B. Boucher, M.P.G Koopmans, J.J.A van Kampen, S.D. Pas. **Radboudumc, Nijmegen:***HIV treating physicians:* S.S.V. Henriet, M. van de Flier, K. van Aerde. *HIV nurse consultants:* R. Strik-Albers. *HIV clinical virologists/chemists:* J. Rahamat-Langendoen, F.F. Stelma. **Universitair Medisch Centrum Groningen, Groningen:***HIV treating physicians:* E.H. Schölvinck. *HIV nurse consultants:* H. de Groot-de Jonge. *HIV clinical virologists/chemists:* H.G.M. Niesters, C.C. van Leer-Buter, M. Knoester. **Wilhelmina Kinderziekenhuis, UMC Utrecht, Utrecht:***HIV treating physicians:* L.J. Bont, S.P.M. Geelen, T.F.W. Wolfs. *HIV nurse consultants:* N. Nauta. *HIV clinical virologists/chemists:* R. Schuurman, F. Verduyn-Lunel, A.M.J. Wensing.

**COORDINATING CENTRE**

*Director:* P. Reiss. *Deputy director:* S. Zaheri. *Data analysis:* D.O. Bezemer, A.I. van Sighem, C. Smit, F.W.M.N. Wit. *Data management and quality control:* M. Hillebregt, A. de Jong, T. Woudstra. *Data monitoring:* D. Bergsma, S. Grivell, R. Meijering, M. Raethke, T. Rutkens. *Data collection:* L. de Groot, M. van den Akker, Y. Bakker, M. Bezemer, A. El Berkaoui, J. Geerlinks, J. Koops, E. Kruijne, C. Lodewijk, E. Lucas, R. van der Meer, L. Munjishvili, F. Paling, B. Peeck, C. Ree, R. Regtop, Y. Ruijs, L. van de Sande, M. Schoorl, P. Schnörr, E. Tuijn, L. Veenenberg, S. van der Vliet, A. Wisse, E.C. Witte. *Patient registration:* B. Tuk.

**Poland:** Polish paediatric cohort: Head of the team: Prof Magdalena Marczyńska, MD, PhD Members of the team: Jolanta Popielska, MD, PhD; Maria Pokorska-Śpiewak, MD, PhD; Agnieszka Ołdakowska, MD, PhD; Konrad Zawadka, MD, PhD; Urszula Coupland, MD, PhD Administration assistant: Małgorzata Doroba. Affiliation: Medical University of Warsaw, Poland, Department of Children's Infectious Diseases; Hospital of Infectious Diseases in Warsaw, Poland.

**Romania:** ‘Victor Babes’ Hospital Cohort, Bucharest: Dr Luminita Ene.

**Russia:** Federal State-owned Institution ‘Republican Clinical Infectious Diseases Hospital’ of the Ministry of Health of the Russian Federation, St Petersburg: Liubov Okhonskaia, Evgeny Voronin, Milana Miloenko, Svetlana Labutina

**Spain:** CoRISPE-cat, Catalonia: financial support for CoRISPE-cat was provided by the Instituto de Salud Carlos III through the Red Temática de Investigación Cooperativa en Sida. Members: Hospital Universitari Vall d’Hebron, Barcelona (Pere Soler-Palacín, Maria Antoinette Frick and Santiago Pérez-Hoyos (statistician)), Hospital Universitari del Mar, Barcelona (Antonio Mur, Núria López), Hospital Universitari Germans Trias i Pujol, Badalona (María Méndez), Hospital Universitari JosepTrueta, Girona (Lluís Mayol), Hospital Universitari Arnau de Vilanova, Lleida (Teresa Vallmanya), Hospital Universitari Joan XXIII, Tarragona (Olga Calavia), Consorci Sanitari del Maresme, Mataró (Lourdes García), Hospital General de Granollers (Maite Coll), Corporació Sanitària Parc Taulí, Sabadell (Valentí Pineda), Hospital Universitari Sant Joan, Reus (Neus Rius), Fundació Althaia, Manresa (Núria Rovira), Hospital Son Espases, Mallorca (Joaquín Dueñas) and Hospital Sant Joan de Déu, Esplugues (Anna Gamell, Clàudia Fortuny, Antoni Noguera-Julian).

**Spain:** CoRISPE-S and Madrid cohort: María José Mellado, Luis Escosa, Milagros García Hortelano, Talía Sainz (Hospital La Paz);María Isabel González- Tomé, Pablo Rojo, Daniel Blázquez (Hospital Doce de Octubre, Madrid); José Tomás Ramos (Hospital Clínico San Carlos, Madrid); Luis Prieto, Sara Guillén (Hospital de Getafe); María Luisa Navarro, Jesús Saavedra, Mar Santos, Mª Angeles Muñoz, Beatriz Ruiz, Carolina Fernandez Mc Phee, Santiago Jimenez de Ory,Susana Alvarez (Hospital Gregorio Marañón); Miguel Ángel Roa (Hospital de Móstoles); José Beceiro (Hospital Príncipe de Asturias, Alcalá de Henares); Jorge Martínez (Hospital Niño Jesús, Madrid); Katie Badillo (Hospital de Torrejón); Miren Apilanez (Hospital de Donostia, San Sebastián); Itziar Pocheville (Hospital de Cruces, Bilbao); Elisa Garrote (Hospital de Basurto, Bilbao); Elena Colino (Hospital Insular Materno Infantil, Las Palmas de Gran Canaria); Jorge Gómez Sirvent (Hospital Virgen de la Candelaria, Santa Cruz de Tenerife); Mónica Garzón, Vicente Román (Hospital de Lanzarote); Abián Montesdeoca, Mercedes Mateo (Complejo Universitario de Canarias, La Laguna-Tenerife),María José Muñoz, Raquel Angulo (Hospital de Poniente, El Ejido); Olaf Neth, Lola Falcón (Hospital Virgen del Rocio, Sevilla); Pedro Terol (Hospital Virgen de la Macarena, Sevilla); Juan Luis Santos (Hospital Virgen de las Nieves, Granada); David Moreno (Hospital Carlos Haya, Málaga); Francisco Lendínez (Hospital de Torrecárdenas, Almería); Ana Grande (Complejo Hospitalario Universitario Infanta Cristina, Badajoz); Francisco José Romero (Complejo Hospitalario de Cáceres); Carlos Pérez (Hospital de Cabueñes, Gijón); Miguel Lillo (Hospital de Albacete); Begoña Losada (Hospital Virgen de la Salud, Toledo); Mercedes Herranz (Hospital Virgen del Camino, Pamplona); Matilde Bustillo, Carmelo Guerrero (Hospital Miguel Servet, Zaragoza); Pilar Collado (Hospital Clínico Lozano Blesa, Zaragoza); José Antonio Couceiro (Complejo Hospitalario de Pontevedra); Amparo Pérez, Ana Isabel Piqueras, Rafael Bretón, Inmaculada Segarra (Hospital La Fe, Valencia); César Gavilán (Hospital San Juan de Alicante); Enrique Jareño (Hospital Clínico de Valencia); Elena Montesinos (Hospital General de Valencia); Marta Dapena (Hospital de Castellón); Cristina Álvarez (Hospital Marqués de Valdecilla, Santander); Ana Gloria Andrés (Hospital de León); Víctor Marugán, Carlos Ochoa (Hospital de Zamora); Santiago Alfayate, Ana Isabel Menasalvas (Hospital Virgen de la Arrixaca, Murcia); Elisa de Miguel (Complejo Hospitalario San Millán-San Pedro, Logroño) and Paediatric HIV-BioBank integrated in the Spanish AIDS Research Network and collaborating Centers.

**Funding:** This work has been partially funded by the Fundación para la Investigación y Prevención de SIDA en España (FIPSE) (FIPSE 3608229/09, FIPSE 240800/09, FIPSE 361910/10), Red Temática de Investigación en SIDA (RED RIS) supported by Instituto de Salud Carlos III (ISCIII) (RD12/0017/0035 and RD12/0017/0037), project as part of the Plan R+D+I and cofinanced by ISCIII- Subdirección General de Evaluación and Fondo Europeo de Desarrollo Regional (FEDER),Mutua Madrileña 2012/0077, Gilead Fellowship 2013/0071, FIS PI15/00694,CoRISpe (RED RIS RD06/0006/0035 y RD06/0006/0021).

**Sweden:** Karolinska University Hospital, Stockholm (Lars Naver, Sandra Soeria-Atmadja, Vendela Hagås).

**Switzerland:***Members of the Swiss HIV Cohort Study (SHCS) and the Swiss Mother and Child HIV Cohort Study:* Aebi-Popp K, Anagnostopoulos A, Asner S, Battegay M, Baumann M, Bernasconi E, Böni J, Braun DL, Bucher HC, Calmy A, Cavassini M, Ciuffi A, Duppenthaler A, Dollenmaier G, Egger M, Elzi L, Fehr J, Fellay J, Francini K, Furrer H, Fux CA, Grawe C, Günthard HF (President of the SHCS), Haerry D (deputy of ‘Positive Council’), Hasse B, Hirsch HH, Hoffmann M, Hösli I, Huber M, Kahlert CR (Chairman of the Mother & Child Substudy), Kaiser L, Keiser O, Klimkait T, Kottanattu L, Kouyos RD, Kovari H, Ledergerber B, Martinetti G, Martinez de Tejada B, Marzolini C, Metzner KJ, Müller N, Nicca D, Paioni P, Pantaleo G, Perreau M, Polli Ch, Rauch A (Chairman of the Scientific Board), Rudin C, Scherrer AU (Head of Data Centre), Schmid P, Speck R, Stöckle M (Chairman of the Clinical and Laboratory Committee), Tarr P, Thanh Lecompte M, Trkola A, Vernazza P, Wagner N, Wandeler G, Weber R, Wyler CA, Yerly S. *Funding:* This study has been financed within the framework of the Swiss HIV Cohort Study, supported by the Swiss National Science Foundation (grant #177499).

**Thailand:** Program for HIV Prevention & Treatment (PHPT). Participating hospitals: Lamphun: Pornpun Wannarit; Phayao Provincial Hospital: Pornchai Techakunakorn; Chiangrai Prachanukroh: Rawiwan Hansudewechakul; Chiang Kham: Vanichaya Wanchaitanawong; Phan: Sookchai Theansavettrakul; Mae Sai: Sirisak Nanta; Prapokklao: Chaiwat Ngampiyaskul; Banglamung: Siriluk Phanomcheong; Chonburi: Suchat Hongsiriwon; Rayong: Warit Karnchanamayul; Bhuddasothorn Chacheongsao: Ratchanee Kwanchaipanich; Nakornping: Suparat Kanjanavanit; Somdej Prapinklao: Nareerat Kamonpakorn, Maneeratn Nantarukchaikul; Bhumibol Adulyadej: Prapaisri Layangool, Jutarat Mekmullica; Pranangklao: Paiboon Lucksanapisitkul, Sudarat Watanayothin; Buddhachinaraj: Narong Lertpienthum; Hat Yai: Boonyarat Warachit; Regional Health Promotion Center 6, Khon Kaen: Sansanee Hanpinitsak; Nong Khai: Sathit Potchalongsin; Samutsakhon: Pimpraphai Thanasiri, Sawitree Krikajornkitti; Phaholpolphayuhasena: Pornsawan Attavinijtrakarn; Kalasin: Sakulrat Srirojana; Nakhonpathom: Suthunya Bunjongpak; Samutprakarn: Achara Puangsombat; Mahasarakam: Sathaporn Na-Rajsima; Roi-et: Pornchai Ananpatharachai; Sanpatong: Noppadon Akarathum; Vachira Phuket: Weerasak Lawtongkum; Chiangdao: Prapawan Kheunjan, Thitiporn Suriyaboon, Airada Saipanya.

Data management team: Kanchana Than-in-at, Nirattiya Jaisieng, Rapeepan Suaysod, Sanuphong Chailoet, Naritsara Naratee, and Suttipong Kawilapat.

**UK & Ireland:** Collaborative HIV Paediatric Study (CHIPS): CHIPS is funded by the NHS (London Specialised Commissioning Group) and has received additional support from Bristol-Myers Squibb, Boehringer Ingelheim, GlaxoSmithKline, Roche, Abbott, and Gilead Sciences. The MRC Clinical Trials Unit at UCL is supported by the Medical Research Council (https://www.mrc.ac.uk) programme number MC_UU_12023/26.

**CHIPS Steering Committee:** Hermione Lyall (chair), Alasdair Bamford, Karina Butler, Katja Doerholt, Conor Doherty, Caroline Foster, Kate Francis, Ian Harrison, Julia Kenny, Nigel Klein, Gillian Letting, Paddy McMaster, Fungai Murau, Edith Nsangi, Helen Peters, Katia Prime, Andrew Riordan, Fiona Shackley, Delane Shingadia, Sharon Storey, Claire Thorne, Gareth Tudor-Williams, Anna Turkova, Steve Welch. **MRC Clinical Trials Unit:** Intira Jeannie Collins, Claire Cook, Siobhan Crichton, Donna Dobson, Keith Fairbrother, Diana M. Gibb, Lynda Harper, Ali Judd, Marthe Le Prevost, Nadine Van Looy. **National Study of HIV in Pregnancy and Childhood, UCL:** Helen Peters, Claire Thorne.

**Participating hospitals:** Republic of Ireland: Our Lady's Children's Hospital Crumlin, Dublin: K Butler, A Walsh. UK: University Hospitals Birmingham NHS Foundation Trust, Birmingham: L Thrasyvoulou, S Welch; University Hospitals Bristol NHS Foundation Trust, Bristol: J Bernatoniene, F Manyika; Calderdale and Huddersfield NHS Foundation Trust, Halifax: G Sharpe; Derby Teaching Hospitals NHS Foundation Trust: B Subramaniam; Middlesex: K Sloper; East Sussex Healthcare NHS Trust, Eastbourne: K Fidler, Glasgow Royal Hospital for Children, Glasgow: R Hague, V Price; Great Ormond Street Hospital for Children NHS Foundation Trust, London: M Clapson, J Flynn, A Cardoso M Abou – Rayyah, N Klein, D Shingadia; Homerton University Hospital NHS Foundation Trust, London: D Gurtin, Oxford University Hospitals NHS Foundation Trust, Oxford: S Yeadon, S Segal; King's College Hospital NHS Foundation Trust, London: C Ball, S Hawkins; Leeds Teaching Hospitals NHS Trust, Leeds: M Dowie; University Hospitals of Leicester NHS Trust, Leicester: S Bandi, E Percival; Luton and Dunstable Hospital NHS Foundation Trust, Luton: M Eisenhut; K Duncan, S Clough; Milton Keynes General University Hospital NHS Foundation Trust, Milton Keynes: Dr L Anguvaa, S Conway, Newcastle upon Tyne Hospitals NHS Foundation Trust, Newcastle: T Flood, A Pickering; The Pennine Acute Hospitals NHS Trust, Manchester: P McMaster C Murphy; North Middlesex University Hospital NHS Trust, London: J Daniels, Y Lees; Northampton General Hospital NHS Trust, Northampton: F Thompson; London North West Healthcare NHS Trust, Middlesex; B Williams, S Pope; Nottingham University Hospitals NHS Trust, Nottingham: L Cliffe, A Smyth, S Southall; Portsmouth Hospitals NHS Trust, Portsmouth: A Freeman; Raigmore Hospital, Inverness: H Freeman; Royal Belfast Hospital for Sick Children, Belfast: S Christie; Royal Berkshire NHS Foundation Trust, Reading: A Gordon; Royal Children's Hospital, Aberdeen: D Rogahn L Clarke; Royal Edinburgh Hospital for Sick Children, Edinburgh: L Jones, B Offerman; Royal Free NHS Foundation Trust, London: M Greenberg; Alder Hey Children's NHS Foundation Trust, Liverpool: C Benson, A Riordan; Sheffield Children's NHS Foundation Trust, Sheffield: L Ibberson, F Shackley; University Hospital Southampton NHS Foundation Trust, Southampton: SN Faust, J Hancock; St George's University Hospitals NHS Foundation Trust, London: K Doerholt, K Prime, M Sharland, S Storey; Imperial College Healthcare NHS Trust, London: H Lyall, C Monrose, P Seery, G Tudor-Williams; Guy's and St Thomas’ NHS Foundation Trust, London:, E Menson, A Callaghan; University Hospitals of North Midlands NHS Trust, Stoke On Trent: A Bridgwood, P McMaster; University Hospital of Wales, Cardiff: J Evans, E Blake; NHS Frimley Health Foundation Trust, Slough: A Yannoulias.

Funding: EPPICC receives funding from the PENTA Foundation (http://penta-id.org). The MRC Clinical Trials Unit at UCL is supported by the Medical Research Council (programme number MC_UU_12023/26).

### 5.1 Conflicts of interest

There are no conflicts of interest.

## Supplementary Material

Supplemental Digital Content

## Figures and Tables

**Table 1:** Characteristics of 1094 young people living with HIV at antiretroviral therapy initiation stratified by height-for age *z*-scores.

Height-for-age *z*-score at ART initiation						
*N* (%) or median (IQR)	All	<−3 SD (Severely stunted)	−3 to <−2 SD (Stunted)	−2 to <−1 SD	≥−1 SD	*P*
All	1094	157 (14)	187 (17)	272 (25)	478 (44)	
Male	526 (48)	83 (53)	90 (48)	117 (43)	236 (49)	0.207
Country						
UK and Ireland	517 (47)	17 (11)	56 (30)	137 (50)	307 (64)	<0.001
Thailand	352 (32)	121 (77)	103 (55)	84 (31)	44 (9)	
Other^a^	225 (21)	19 (12)	28 (15)	51 (19)	127 (27)	
Ethnicity						
White	99 (9)	10 (6)	12 (6)	26 (10)	51 (11)	<0.001
Black	484 (44)	16 (10)	49 (26)	125 (46)	294 (62)	
Asian	365 (33)	121 (77)	106 (57)	89 (33)	49 (10)	
Other	63 (6)	6 (4)	5 (3)	19 (7)	33 (7)	
Unknown/Prohibited	83 (8)	4 (3)	15 (8)	13 (5)	51 (11)	
Born abroad	370 (35)	21 (14)	51 (28)	100 (37)	198 (43)	<0.001
Age						
Median years	6.4 (2.8–9.0)	6.4 (2.7–9.0)	7.1 (3.8–9.5)	6.4 (3.2–8.7)	6.1 (2.5–8.8)	<0.001
1 to 2 years	280 (26)	45 (29)	35 (19)	65 (24)	135 (28)	
3 to 5 years	237 (22)	31 (20)	43 (23)	64 (24)	99 (21)	
6 to 10 years	577 (53)	81 (52)	109 (58)	143 (53)	244 (51)	
Year started ART						
Median (IQR)	2004 (2003–2007)	2003 (2003–2008)	2004 (200–2006)	2004 (2003–2006)	2005 (2003–2008)	<0.001
<2004	433 (40)	79 (50)	78 (42)	116 (43)	160 (33)	
2004–2007	445 (41)	69 (44)	93 (50)	106 (39)	177 (37)	
≥2008	216 (20)	9 (6)	16 (9)	50 (18)	141 (30)	
NNRTI-based regimen	880 (80)	141 (90)	167 (89)	222 (82)	350 (73)	<0.001
Viral load						
Value present	980 (90)	133 (85)	167 (89)	241 (89)	439 (92)	
Median log VL	5.0 (4.5–5.5)	5.3 (4.9–5.7)	5.0 (4.7–5.5)	5.0 (4.5–5.4)	5.0 (4.2–5.5)	<0.001
≤400	36 (4)	4 (3)	4 (2)	6 (2)	22 (5)	
>400 to 1000	13 (1)	1 (1)	1 (1)	1 (0)	10 (2)	
>1000–10 000	91 (9)	7 (5)	11 (7)	21 (9)	52 (12)	
>10 000–100 000	435 (35)	39 (29)	60 (36)	89 (27)	157 (36)	
>100 000	495 (51)	82 (62)	91 (54)	124 (51)	198 (45)	
WHO immunological classification						
Value present	1006 (92)	145 (92)	177 (95)	258 (95)	426 (89)	
None or not significant	164 (16)	7 (5)	16 (9)	40 (16)	101 (24)	<0.001
Mild	110 (11)	5 (3)	12 (7)	27 (10)	66 (15)	
Advanced	129 (13)	6 (4)	12 (7)	35 (14)	76 (18)	
Severe	603 (60)	127 (88)	137 (77)	156 (60)	183 (43)	
zBMI						
Value present	1089 (100)	157 (100)	186 (100)	271 (100)	475 (99)	
Median zBMI	−0.1 (−1.1 to 0.8)	−1.1 (−2.3 to 0.2)	−0.7 (−1.6 to 0.3)	−0.1 (−0.9 to 0.7)	0.3 (−0.5 to 1.1)	<0.001
<−2 SD (Thinness)	133 (12)	49 (31)	34 (18)	22 (8)	28 (6)	
−2 to <−1 SD	164 (15)	32 (20)	42 (23)	42 (16)	48 (10)	
−1 to <1 SD	576 (53)	59 (38)	96 (62)	158 (58)	263 (55)	
1 to <2 SD (Overweight)	160 (15)	13 (8)	12 (6)	37 (14)	98 (21)	
≥2 SD (Obese)	56 (5)	4 (3)	2 (1)	12 (4)	38 (8)	

IQR, interquartile range; VL, viral load; zBMI, BMI-for-age *z*-scores.

^a^Other includes Belgium, Greece, Latvia, Netherlands, Poland, Romania, Spain, Sweden and Switzerland.

**Table 2:** Association between characteristics at antiretroviral therapy initiation and average height, timing and shape of growth spurt after adjustment for baseline age and height-for-age *z*-score in 918 young people living with HIV.

	Average height			Timing of growth spurt			Shape of growth spurt		
	coef	95% CI	*P*	coef	95% CI	*P*	coef	95% CI	*P*
Girls	0.58	−0.18 to 1.34	0.132	0.04	−0.08 to 0.16	0.485	0.007	−0.015 to 0.029	0.555
Country (ref: Other Europe)									
Thailand	−3.06	−4.34 to −1.78	<0.001	0.04	−0.16 to 0.24	0.701	−0.030	−0.067 to 0.007	0.116
UK & Ireland	−0.55	−1.65 to 0.55	0.325	0.10	−0.07 to 0.29	0.241	−0.033	−0.065 to −0.001	0.044
Year (ref: <2004)									
2004 to <2007	−0.31	−1.17 to 0.55	0.476	0.00	−0.14 to 0.14	0.991	0.010	−0.015 to 0.035	0.426
≥2008	−0.22	−1.33 to 0.90	0.704	−0.08	−0.26 to 0.10	0.369	0.011	−0.022 to 0.043	0.512
NNRTI based regimen (ref: PI-based regimen)	0.27	−0.78 to 1.31	0.616	−0.04	−0.20 to 0.13	0.673	−0.018	−0.048 to 0.012	0.240
Viral load (per log increase)	−0.10	−0.51 to 0.30	0.615	0.03	−0.03 to 0.10	0.292	−0.002	−0.013 to 0.010	0.787
WHO immunological classification (ref: None)									
Mild	−0.10	−1.56 to 1.35	0.891	0.06	−0.17 to 0.29	0.608	−0.003	−0.045 to 0.039	0.900
Advanced	−0.56	−1.96 to 0.84	0.433	−0.06	−0.29 to 0.16	0.581	−0.016	−0.057 to 0.024	0.426
Severe	0.28	−0.84 to 1.39	0.628	−0.01	−0.18 to 0.17	0.948	−0.002	−0.034 to 0.031	0.927
zBMI (per 1SD increase)	−0.32	−0.60 to −0.05	0.021	−0.07	−0.11 to −0.02	0.003	−0.007	−0.015 to 0.001	0.083

Individual size, tempo and velocity parameters were estimated using the SITAR model described in the results and table S1 and represent the differences in size, tempo and velocity unexplained by age and HAZ at ART initiation. Model included data from 918 of the 1094 children included in the SITAR model for which data on the explanatory variables were complete. CI, confidence interval; NNRTI, nonnucleoside reverse transcriptase inhibitor; PI, protease inhibitor; zBMI, BMI-for-age *z*-scores.
