# Supplementary material for: Height and timing of growth spurt during puberty in young people living with vertically acquired HIV in Europe and Thailand
Source: AIDS. 2019 Jul 9;33(12):1897–910. doi: 10.1097/QAD.0000000000002294 (PMC6738540; doi:10.1097/QAD.0000000000002294)
Supplement: Supplemental Digital Content [file aids-33-1897-s001.doc]

Table S1: Characteristics of 1943 young people living with HIV who did and did not have the required height data in order to be included in the models

| N(%) or median[IQR] | Included in models | Not included in models | p-value | Missing baseline height | No height data after age 8 |
| --- | --- | --- | --- | --- | --- |
| **All** | 1094 | 849 |  | 721 | 202 |
| **Male** | 526(48) | 409(48) | 0.947 | 359(50) | 89(44) |
| **Country** |  |  |  |  |  |
| UK & Ireland | 517(47) | 346(41) | <0.001 | 324(45) | 54(27) |
| Thailand | 352(32) | 209(25) |  | 146(20) | 72(36) |
| Other | 225(21) | 294(35) |  | 251(35) | 76(38) |
| **Ethnicity** |  |  |  |  |  |
| White | 99(9) | 111(13) | <0.001 | 96(13) | 26(13) |
| Black | 484(44) | 384(45) |  | 350(49) | 66(33) |
| Asian | 365(33) | 218(26) |  | 154(21) | 74(37) |
| Other | 63(6) | 52(6) |  | 48(7) | 6(3) |
| Prohibited | 63(6) | 59(7) |  | 48(7) | 26(13) |
| Unknown | 20(2) | 25(3) |  | 24(3) | 4(2) |
| **Born abroad** | 370(35) | 373 (44) | <0.001 | 317(47) | 67(35) |
| **Age at ART initiation (years)** |  |  |  |  |  |
| Median[IQR] | 6.4[2.8,9.0] | 3.7[0.9,7.1] | <0.001 | 4.2[0.9,7.7] | 1.5[0.5,4.0] |
| **Initial NNRTI based ART regimen** | 880(80) | 643(76) | 0.012 | 556(77) | 137(68) |
| **Year started ART** |  |  |  |  |  |
| Median[IQR] | 2004[2003,2007] | 2005[2003,2007] | 0.044 | 2004[2003,2007] | 2006[2003,2009] |
| **Viral load at ART initiation** |  |  |  |  |  |
| Value present | 980 | 411 |  | 295 | 155 |
| Median log VL | 5.0[4.5,5.5] | 5.3[4.7,5.7] | <0.001 | 5.3[4.7,5.7] | 5.4[4.9,5.8] |
| **WHO immunological classification** |  |  |  |  |  |
| Value present | 1006 | 446 |  | 326 | 155 |
| None or not significant | 164(16) | 91(20) | 0.041 | 69(21) | 32(21) |
| Mild | 110(11) | 63(14) |  | 48(15) | 16(10) |
| Advanced | 129(13) | 56(13) |  | 43(13) | 19(12) |
| Severe | 603(60) | 236(53) |  | 166(51) | 88(57) |
| **zBMI at ART initiation** |  |  |  |  |  |
| Value present | 1089 |  |  |  | 135 |
| Median zBMI | -0.1[-1.1,0.8] |  |  |  | -0.4[-1.3,0.5] |
| **HAZ at ART initiation** |  |  |  |  |  |
| Value present | 1094 |  |  |  | 137 |
| Median HAZ | -1.2[-2.3,-0.2] |  |  |  | -1.5[-2.7,-0.6] |

Table S2: Actual height at age 16 years of 463 young people living with HIV in paediatric care at 16 years by Height-for-age z scores and age at ART initiation

|  | **Height at age 16 (cm)** | | | |
| --- | --- | --- | --- | --- |
|  | Boys, N = 229 | | Girls, N = 234 | |
|  | mean(sd) | p-value | mean(sd) | p-value |
| **All** | 166(8.7) |  | 158(6.9) |  |
| **Age at ART initiation** |  |  |  |  |
| 1 to 2 years | 168(6.7) | <0.001 | 160(6.5) | <0.001 |
| 3 to 5 years | 168(8.0) |  | 159(6.6) |  |
| 6 to 10 years | 165(8.9) |  | 157(6.9) |  |
| **HAZ at ART initiation** |  |  |  |  |
| <-3 SD (Severe stunting) | 154(8.1) | <0.001 | 152(5.3) | <0.001 |
| -3 to <-2 SD (Stunting) | 161(5.5) |  | 154(6.5) |  |
| -2 to <-1 SD | 164(5.2) |  | 157(3.9) |  |
| ≥-1 SD | 172(6.1) |  | 162(6.5) |  |
| **WHO reference height** | 173(7.8) |  | 163(6.8) |  |

Table S3: Results of SITAR models for height from age 8 to 19 years in 526 boys and 568 girls living with HIV

|  | **Average height** | | |  | **Timing of growth spurt** | | |  | **Shape of growth spurt** | | |
| --- | --- | --- | --- | --- | --- | --- | --- | --- | --- | --- | --- |
|  | **coef** | **95% CI** | **p-value** |  | **coef** | **95% CI** | **p-value** |  | **coef** | **95% CI** | **p-value** |
| **Males** |  |  |  |  |  |  |  |  |  |  |  |
| HAZ at ART initiation (ref: ≥-1) |  |  |  |  |  |  |  |  |  |  |  |
| -2 to <-1 SD | -7.86 | -11.96, -3.76 | <0.001 |  | -0.51 | -1.20, 0.17 | 0.141 |  | -0.01 | -0.05, 0.03 | 0.600 |
| -3 to <-2 SD | -5.60 | -9.50, -1.69 | 0.005 |  | -0.39 | -1.03, 0.26 | 0.239 |  | 0.01 | -0.04, 0.05 | 0.722 |
| <-3 SD | -5.59 | -9.30, -1.89 | 0.003 |  | 0.11 | -0.51, 0.73 | 0.727 |  | 0.01 | -0.04, 0.06 | 0.722 |
| Age at ART initiation (ref: 1 to 2) |  |  |  |  |  |  |  |  |  |  |  |
| 3 to 5 years | -0.67 | -3.51, 2.17 | 0.644 |  | -0.29 | -0.76, 0.18 | 0.231 |  | 0.01 | -0.03, 0.06 | 0.564 |
| 6 to 10 years | 0.47 | -1.97, 2.91 | 0.706 |  | 0.06 | -0.35, 0.46 | 0.783 |  | 0.04 | 0.00, 0.08 | 0.067 |
| Interaction (HAZ x AGE) |  |  |  |  |  |  |  |  |  |  |  |
| HAZ -2 to <-1 SD & age 3 to 5 | 0.95 | -4.08, 5.99 | 0.711 |  | 0.36 | -0.47, 1.19 | 0.399 |  |  |  |  |
| HAZ -3 to <-2 SD & age 3 to 5 | -3.49 | -8.93, 1.95 | 0.209 |  | 0.79 | -0.11, 1.68 | 0.086 |  |  |  |  |
| HAZ <-3 SD & age 3 to 5 | -10.28 | -15.84, -4.72 | <0.001 |  | 0.15 | -0.77, 1.07 | 0.754 |  |  |  |  |
| HAZ -2 to <-1 SD & age 6 to 10 | 1.66 | -2.98, 6.30 | 0.485 |  | 0.96 | 0.19, 1.72 | 0.015 |  |  |  |  |
| HAZ -3 to <-2 SD & age 6 to 10 | -7.35 | -11.87, -2.84 | 0.001 |  | 0.92 | 0.17, 1.66 | 0.016 |  |  |  |  |
| HAZ <-3 SD & age 6 to 10 | -11.72 | -16.17, -7.27 | <0.001 |  | 0.42 | -0.32, 1.16 | 0.267 |  |  |  |  |
| **Females** |  |  |  |  |  |  |  |  |  |  |  |
| HAZ at ART initiation (ref: ≥-1) |  |  |  |  |  |  |  |  |  |  |  |
| -2 to <-1 SD | -1.21 | -3.14, 0.73 | 0.222 |  | 0.48 | 0.27, 0.69 | <0.001 |  | -0.01 | -0.06, 0.03 | 0.489 |
| -3 to <-2 SD | -2.29 | -5.12, 0.53 | 0.112 |  | 0.73 | 0.48, 0.97 | <0.001 |  | 0.02 | -0.03, 0.07 | 0.367 |
| <-3 SD | -5.22 | -7.91, -2.53 | <0.001 |  | 1.50 | 1.21, 1.78 | <0.001 |  | -0.03 | -0.08, 0.03 | 0.334 |
| Age at ART initiation (ref: 1 to 2) |  |  |  |  |  |  |  |  |  |  |  |
| 3 to 5 years | 2.04 | -0.01, 4.08 | 0.051 |  | 0.03 | -0.24, 0.29 | 0.859 |  | 0.04 | -0.01, 0.09 | 0.113 |
| 6 to 10 years | 2.54 | 0.94, 4.13 | 0.002 |  | 0.41 | 0.20, 0.62 | 0.002 |  | 0.08 | 0.04, 0.12 | 0.002 |
| Interaction (HAZ x AGE) |  |  |  |  |  |  |  |  |  |  |  |
| HAZ -2 to <-1 SD & age 3 to 5 | -3.84 | -6.78, -0.90 | 0.011 |  |  |  |  |  |  |  |  |
| HAZ -3 to <-2 SD & age 3 to 5 | -4.75 | -8.34,-1.15 | 0.010 |  |  |  |  |  |  |  |  |
| HAZ <-3 SD & age 3 to 5 | -0.79 | -4.48, 2.90 | 0.676 |  |  |  |  |  |  |  |  |
| HAZ -2 to <-1 SD & age 6 to 10 | -5.50 | -7.71, -3.28 | <0.001 |  |  |  |  |  |  |  |  |
| HAZ -3 to <-2 SD & age 6 to 10 | -8.08 | -11.15, -5.01 | <0.001 |  |  |  |  |  |  |  |  |
| HAZ <-3 SD & age 6 to 10 | -9.12 | -12.13, -6.11 | <0.001 |  |  |  |  |  |  |  |  |

Note: SITAR regression with changes in height over time modelled using a natural cubic spline with 6 knots, used to estimate differences in size, tempo and velocity associated with age and HAZ at ART initiation. Separate models were fitted to males and females
